# A Pollution Detection System for Plastic Ocean Waste Based on Energy-Harvesting Radio Transmitters

**DOI:** 10.3390/s26103090

**Published:** 2026-05-13

**Authors:** Vitalii Beschastnyi, Darya Ostrikova, Konstantin Samouylov

**Affiliations:** Department of Probability Theory and Cybersecurity, Peoples’ Friendship University of Russia (RUDN University), 117198 Moscow, Russia; ostrikova-dyu@rudn.ru (D.O.); samuylov-ke@rudn.ru (K.S.)

**Keywords:** energy harvesting, ultra-low-power, ultra-low-frequency, wireless sensor networks, ocean pollution

## Abstract

With the constant increase in the usage of plastic bottles in food production, ocean pollution has become a significant problem. The ability to organize in large fields is one of the critical problems nowadays, and their detection for further removal is a challenge. In this study, we propose the idea of equipping some of the plastic bottles on the production lines with simple radio-emitting equipment capable of signaling the presence of plastic bottle fields in the ocean to nearby vessels. The proposed idea is based on ultra-low-power energy harvesting that utilizes inherent wave energy. To assess the performance of the proposed framework, we developed a performance evaluation framework that captures the main specifics of the proposed detection system, including the probability of detecting at least one waste field and all waste fields in a given region. To showcase the potential of the proposed idea in this study, we also demonstrate that ultra-low-power harvesting using ocean waves is feasible. Our numerical results illustrate that for typical environmental parameters, the time range for detecting all waste fields in the area scales from 4–6 h to a few days at most. Additionally, the probability of detecting the presence of waste in the area is 2–3 times higher, potentially allowing for extremely fast detection and timely removal. We emphasize that the proposed system can be used to complement the currently available systems, not to replace them completely.

## 1. Introduction

Human activities have established pollution as a major threat to global ecological stability. Over the last century, accelerated industrialization, demographic expansion, and intensifying urbanization have catalyzed pollutant accumulation across terrestrial, atmospheric, and aquatic biomes. In this context, marine pollution has emerged as a focal point for scientific and policy concerns, given its profound implications for global biodiversity, regulation of planetary climate systems and preservation of human health [1].

Marine ecosystems are experiencing an accelerating influx of synthetic polymers and chemical effluents [2]. Empirical data indicate that human drivers represent over 80% of the total marine pollution load [3], primarily partitioned between industrial discharge, agrochemical runoff, and mismanaged plastic waste [4,5]. The proliferation of microplastics has emerged as a critical ecological threat. Their environmental persistence is compounded by their ability to accumulate within marine organisms, presenting a significant risk to the human food chain [6].

Despite advancements in marine observation, real-time pollution monitoring is hampered by the immense scale, geographical isolation, and stochastic dynamics of oceanic environments [7]. Current frameworks primarily depend on satellite remote sensing and autonomous platforms [8]. Close-to-the-shore terrestrial connectivity can be used [9] while in open waters—non-terrestrial systems (NTN, [10]). However, satellite-based architectures face significant economic barriers and reliability bottlenecks when processing high-bandwidth, real-time data streams [11]. In the subsurface domain, underwater communications via acoustic signals are strictly limited by environmental interference, which degrades both the transmission range and signal integrity [12]. Furthermore, the operational longevity of autonomous monitoring systems is affected by energy density constraints, as the burden of frequent battery replenishment remains a critical failure point for conventional wireless deployments [13].

The integration of advanced pollution control systems into ocean environments remains a key focus of state-of-the-art research, with several strategies developed and assessed for their effectiveness in mitigating marine pollution. These approaches aim to leverage modern technologies to provide more accurate and timely assessments of oceanic conditions, with financial reasoning in mind. Specifically, this study aims to propose and evaluate a simple yet effective system for detecting plastic fields in oceans for further collection and disposal. To this end, we first provide an overview of current ocean pollution detection systems. We then demonstrate that a low-cost, ultra-low-power, self-harvesting radio transmission system capable of emitting notification signals over large distances can be designed and integrated into plastic bottles. We then developed a performance evaluation framework for the proposed system, which specifies the detection probability as a function of the system design parameters, including the fraction of bottles to be equipped with self-powered radio modules, the frequency of vessels passing close to the waste fields, and the size of the waste field.

The main contributions of this study are as follows:

- review of existing pollution control strategies, their effectiveness, and integration with modern technologies for managing ocean pollution;
- discussion of concepts for energy-harvesting prototype at low frequencies generated by ocean waves that can be utilized to power the radio module;
- new mathematical model designed to enhance real-time monitoring, prediction, and analysis of ocean pollution, accounting for dynamic environmental variables.

We note that our primary objective is to stimulate fresh interest in diversified ocean pollution control. Accordingly, we present a detailed system analysis and a performance evaluation framework to highlight its feasibility, alongside a discussion of the core principles for building an effective energy harvester.

These tools account for dynamic environmental variables, enabling better prediction and analysis of pollutant dispersion and behavior over time. Furthermore, the design and evaluation of new pollution analysis prototypes using innovative metrics show promise for enhancing the detection and impact assessment of ocean pollutants. These tools aim to not only improve current monitoring capabilities but also provide more accurate long-term trend analysis, offering insights that can help inform policy decisions and mitigate the adverse effects of pollution on marine ecosystems.

## 2. Related Work

In this section, we outline the related work. We will begin by discussing the impact of ocean pollution and the role of plastic waste in overall waste. We then cover state-of-the-art pollution detection techniques and methods for its removal.

### 2.1. Plastic Waste Impact

Plastic pollution pervades oceans, lakes, and reservoirs worldwide, harming marine life, altering carbon cycling, and transferring microplastics and toxins into the food chain. The UNEP report [14] notes that marine plastics constitute at least 85% of marine waste, with 75–199 million tons already in the ocean, and annual leakage projected to nearly triple by 2040 to 23–37 million tons. Most plastic originates on land, persists for decades, and causes economic losses of $6–19 billion (2018), rising to $100 billion annually by 2040, plus $80–120 billion lost yearly in packaging value. The global recycling rate is below 10%, and plastic emissions could reach 15% of the carbon budget by 2050.

The authors of [15] reported that approximately 8 million tons of plastic enter oceans annually, harming 914 marine species through ingestion and entanglement, smothering plants such as algae, and degrading habitats such as mangroves. Current control measures include incineration and landfilling, with recycling emphasized as a key strategy.

A global study [16] found plastic debris in all freshwater ecosystems, with some lakes (Lugano and Tahoe) matching oceanic gyre levels. Lakes are vulnerable in densely populated watersheds (textile fibers) or large lakes with long retention times (polypropylene and polyethylene fragments). Fibers appear even in remote lakes via atmospheric transport, making lakes key sinks in the plastic cycle, and requiring their inclusion in management strategies.

### 2.2. Plastic Waste Fields

Plastic waste fields are diffuse “soups” of microplastics suspended below the ocean surface. These fields are primarily formed by gyres, large systems of rotating ocean currents that act as vortexes, drawing in and trapping plastic pollution from coastlines around the Pacific Rim and fishing vessels. Plastic within these accumulation zones is categorized into four classes based on size: microplastics (smaller than 5 mm), mesoplastics, macroplastics, and megaplastics. Remarkably, an estimated 92% of the total mass consists of microplastics [17].

The scale of these oceanic waste fields is enormous. The Great Pacific Garbage Patch covers an area three times the size of France and contains 1.8 trillion pieces [18]. Meanwhile, a South Pacific garbage patch exists off the coasts of Chile and Peru, and the gyre’s currents deposit immense quantities of plastic onto otherwise pristine remote islands with the highest recorded plastic density of up to 37.7 million items [19]. The impact of these immense plastic accumulations is catastrophic, creating a complex web of harm that affects wildlife, ecosystems, human health, and the global economy.

### 2.3. Detection Techniques

The most promising technique for plastic litter detection is remote sensing (RS) [20]. The RS workflow is a multistage process encompassing data acquisition, preprocessing, analysis, validation, mapping, and documentation. During the acquisition phase, high-resolution data are captured using a variety of sensor technologies, including optical, thermal infrared (TIR), hyperspectral (HSI), multispectral (MSI), and synthetic aperture radar (SAR), deployed from satellites, unmanned aerial systems (UAS), and manned aircraft.

Hyperspectral imaging is a highly sophisticated technique that identifies ocean plastic by analyzing unique spectral signatures of materials across the electromagnetic spectrum. Unlike other methods, HSI captures data in hundreds of narrow contiguous bands, providing a continuous spectrum for every pixel in an image. This high spectral resolution allows researchers to perform an in-depth analysis of material composition, making it possible to distinguish between different polymer types of plastic, such as PET (Polyethylene Terephthalate) and HDPE (High-Density Polyethylene), and to differentiate them from natural materials such as seaweed or wood. By mounting hyperspectral sensors on satellites, aircraft, or drones, scientists can create spectral libraries for various plastics and map their distribution over large areas. However, the richness of this data comes with significant challenges, including the generation of massive datasets that require substantial computational power for processing and storage, as well as sensitivity to atmospheric conditions and sunglint that can obscure the water’s surface.

Multispectral imaging is a versatile and widely adopted method for large-scale monitoring of marine plastic pollution, most notably using satellite programs such as Europe’s Sentinel-2. This technique captures image data across a few (typically 3–10) broad spectral bands, focusing on specific wavelengths that are informative for environmental monitoring. Although it lacks the fine spectral details of HSI, MSI effectively distinguishes plastics from other floating materials by detecting their distinct reflectance patterns, particularly in the red, near-infrared, and short-wave infrared regions. Indices such as the Floating Debris Index (FDI) can be calculated from the data to highlight potential plastic accumulations. The primary strengths of the MSI lie in its cost-effectiveness, lower data complexity, and ability to provide extensive spatial and temporal coverage, making it ideal for tracking the movement and buildup of plastic over time. Its limitations include a lower spectral resolution, which can lead to overlapping information with materials such as foam or organic debris, and a continued dependency on clear atmospheric conditions.

Optical imaging is the most fundamental remote sensing method, relying on standard visible light (RGB) sensors to detect plastic debris based on its visual appearance, color, and contrast against the ocean surface. This technology is often deployed on high-resolution satellites and Unmanned Aerial Systems (UAS) or drones to capture detailed photographs of water. Its primary advantage is its ability to provide very high spatial resolution imagery, allowing for the accurate visual identification and classification of larger plastic items. The simplicity and accessibility of optical sensors make them a cost-effective solution for localized surveys and for validating data from more complex systems. Nevertheless, this method is inherently limited by its dependence on sunlight and clear weather; cloud cover, fog, and poor lighting conditions can hinder data collection. Furthermore, because it operates only within the visible spectrum, optical imaging cannot provide detailed material composition information and is only effective for detecting debris floating on the surface.

Thermal Infrared sensing offers a unique approach to plastic detection by measuring the heat emitted by objects, capitalizing on the distinct thermal properties of plastics compared to water. Plastics typically have different thermal emissivity and heat capacity, meaning they warm up and cool down at different rates than the surrounding seawater. TIR sensors can detect these temperature anomalies, creating a thermal image in which plastic litter may appear as a hot or cold field relative to its environment. A significant advantage of this method is its ability to operate independently of sunlight, allowing for effective monitoring at night and through thin cloud cover and providing data that are complementary to optical methods. However, the effectiveness of TIR is highly dependent on the existence of a measurable temperature difference, which may be minimal at certain times of the day or under uniform thermal conditions. It also suffers from a lower spatial resolution, making it difficult to detect small plastic items, and its accuracy can be compromised by factors such as moisture on the plastic or biofouling, which alter thermal characteristics.

Radar remote sensing, particularly using Synthetic Aperture Radar (SAR), is a powerful method for detecting ocean plastic by transmitting microwave signals and analyzing the backscatter that returns from the sea surface. The detection capability is based on the dielectric properties and surface roughness of the materials; plastics influence the scattering of radar waves differently than water or biological matter, producing a unique signal. The paramount advantage of SAR is its independence from weather and sunlight; it can penetrate clouds and operate effectively both day and night, making it ideal for persistent, all-weather monitoring of vast ocean areas. Systems such as those on the Sentinel-1 satellite have been used to identify potential plastic accumulations by analyzing backscatter variations. The main limitations include a generally lower spatial resolution than optical sensors, which can make it challenging to identify small fields of debris, and a lack of spectral information, making it difficult to confirm that a detected target is definitively plastic without corroborating data from other sources. Additionally, its signal can be affected by the sea state and wind conditions.

**Figure 1:**
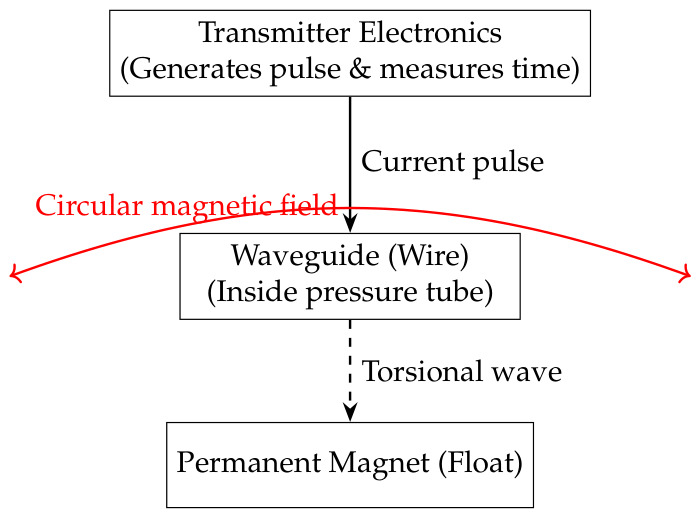
Magnetostrictive transmitter schematic.

In water waste detection, magnetostrictive and inductive transmitters enable a holistic monitoring strategy, with each addressing distinct challenges. Magnetostrictive sensors based on the Wiedemann effect are primarily deployed for continuous, high-precision level measurements in tanks, clarifiers, and digesters. Their schematic comprises a waveguide wire, permanent magnet float, and torsional wave detector (see Figure 1). When applied to wastewater treatment, these sensors monitor sludge levels, chemical tank contents, and water–oil interfaces with an accuracy approaching 0.01% of the full scale [21]. Because the sensing element is non-contact and fully isolated, magnetostrictive transmitters can withstand abrasive slurries, corrosive chemicals, and high humidity, which are typical of treatment plants. Moreover, they can be configured in redundant, power-free loops to ensure level indication even during electrical outages, a critical safety feature for overflow prevention and process continuity [22].

**Figure 2:**
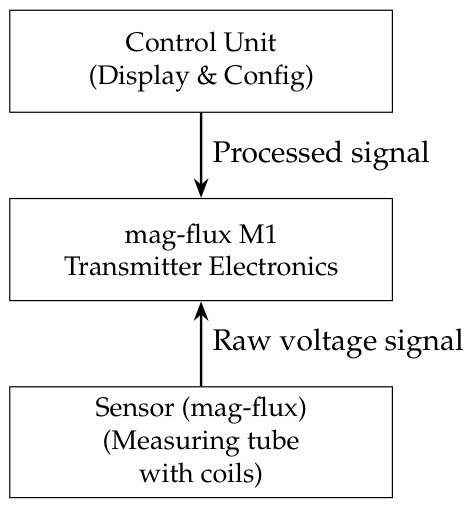
Magnetic-inductive (mag-flux) transmitter.

Inductive transmitters, by contrast, excel in dynamic water flow networks. Their schematic centers on a non-magnetic measuring tube, electromagnetic coils, and sensing electrodes that capture the voltage induced by conductive liquids moving through a magnetic field (Faraday’s law; see Figure 2). In water waste management, these devices function as electromagnetic flow meters for the influent, effluent, and return-activated sludge. They provide real-time flow data with no moving parts, making them immune to clogging and wear. Additionally, inductive conductivity sensors are ideal for monitoring heavily contaminated or scaling wastewater, as their non-contact design resists fouling and polarization [23]. The combination of high accuracy and low maintenance has made inductive transmitters the standard for process control, chemical dosing optimization, and network balancing in modern water resource recovery facilities.

Magnetostrictive sensors watch over static storage and treatment units, ensuring proper levels and preventing spills, while inductive sensors guard the flow paths, measuring volumes and detecting leaks or conductivity anomalies. When integrated into a supervisory control and data acquisition system, they provide operators with the real-time visibility needed to meet discharge permits, reduce energy consumption, and protect receiving waters. Advanced implementations have also used magnetostrictive guided-wave transducers for proactive leak detection in water mains and inductive proximity sensors for sorting metallic debris from wastewater streams before they damage pumps [24]. As water scarcity and stricter regulations intensify, the synergy between magnetostrictive and inductive instrumentation will remain indispensable for resilient and efficient water waste management.

### 2.4. Removal Methods

Marine plastic pollution accumulates on shorelines, sea surfaces, and seabeds. Unlike riverine interception, sea-based removal must contend with open-ocean conditions, dispersed debris, and the fact that an estimated 94% of marine plastic litter has sunk to the seabed [25].

Floating barriers and net-based systems are deployed in coastal waters and oceanic gyres to capture drifting macroplastics. The most prominent example is The Ocean Cleanup’s System 03, which uses a long U-shaped floating barrier towed between two slow-moving vessels. The barrier funnels plastic into a retention zone for periodic extraction and recycling. This system has successfully removed hundreds of tons of plastic from the Great Pacific Garbage Patch; however, it targets only floating debris and cannot recover sunken litter.

To address sunken litter, autonomous and remotely operated robotic systems have been developed. Advanced robotic systems are now being deployed to tackle the most challenging debris, particularly the estimated 94% of litter that sinks to the seabed [25]. Research from the MAELSTROM project demonstrated the efficacy of using acoustic remote sensing and an underwater cable-driven robot to selectively remove macroplastics from the seafloor in the Venice coastal area [26]. This approach successfully collected 2240 kg of marine litter from an urban lagoon site and 260 kg of ropes and floating buoys from an abandoned mussel farm, thereby validating the use of high-resolution multibeam mapping to guide robotic cleanup operations. The SeaClear2.0 project integrates an unmanned surface vessel equipped with multibeam echosounders, an underwater robot (MiniTortuga) with cameras, imaging sonar, and a robotic gripper. In May 2025, SeaClear2.0 autonomously detected, grasped, and lifted a large tyre from the seabed at the Port of Hamburg, achieving positioning errors below one meter and object detection success rates of approximately 80% [27]. These systems are effective but remain costly and are limited to shallow coastal waters (typically <100 m depth).

Unlike macroplastics, microplastics (<5 mm) are dispersed throughout the water column and seabed, making large-scale open-ocean removal impractical. Current sea-based microplastic collection is limited to experimental or monitoring activities, such as manta trawls towed by research vessels to sample surface microplastics. Sediment sieving in coastal areas can extract microbeads and fragments, but no scalable technology exists for the direct extraction of microplastics from the open ocean. Therefore, the effective removal of microplastics depends on their capture in wastewater treatment plants and industrial effluents.

These robotic systems are underpinned by sophisticated mapping algorithms and Artificial Intelligence (AI)-driven detection methods. The authors of [28] developed automated mapping approaches that represent the seafloor as three-dimensional voxel grids, with litter locations identified in camera and sonar images using deep neural networks and projected onto the map through geometric calculations.

Sea-based plastic removal has evolved from manual beach cleanups to autonomous seabed robots. Although surface barriers can capture floating debris in coastal and gyre systems, the vast majority of marine litter lies on the seabed and requires sophisticated robotic intervention. Current robotic systems have proven effective in controlled coastal demonstrations but face limitations in terms of scalability, cost, and depth. Microplastic remediation remains an unresolved challenge in open oceans. Future progress will depend on integrating AI-driven mapping, energy-efficient autonomous platforms, and life cycle thinking to ensure that removal efforts yield a genuine environmental return. Table 1 outlines the principal methods for collecting marine plastic litter.

Satellite-based communication systems are often hindered by high costs and unreliable performance when handling large volumes of real-time data, whereas underwater data transmission technologies face significant operational constraints owing to fluctuating environmental conditions that affect both transmission range and data integrity. These limitations underscore the need for a cost-effective and reliable technique that can accurately detect plastic litter. Compounding this issue is the ongoing challenge of energy dependency in autonomous monitoring platforms, where the need for frequent battery recharging or replacement undermines the feasibility of long-term, sustained data collection and limits the effectiveness of conventional wireless monitoring systems. To address these challenges, litter detection techniques should incorporate energy-harvesting capabilities to enable continuous monitoring across expansive marine areas.

## 3. Considered System

In this section, we first introduce the proposed approach. Then, we define the system modeled in this paper by introducing the deployment, parameters, and metrics of interest. The main notations used for the performance analysis are listed in Table 2.

### 3.1. The Proposed Approach and Rationale

The main idea of the proposed approach consists of utilizing an extremely low-cost radio operating in energy-harvesting mode attached to the bottom of every *i*-th bottle produced. The low cost of the solution is ensured by not utilizing the radio itself for any kind of communications, as only carrier generation at a certain fixed frequency $f_{C}$ is presumed once a sufficient amount of energy is accumulated. Energy harvesting is performed by utilizing ocean waves that are known to fluctuate at low frequencies in the range of 0.05–0.25 Hz, depending on the conditions [29]. It has recently been shown that these ultra-low-frequency vibrations allow for relatively efficient energy harvesters [30,31]; see also Section 5. Vessels are assumed to be equipped with carrier receivers operating at a preconfigured frequency selected for system operation.

**Figure 3:**
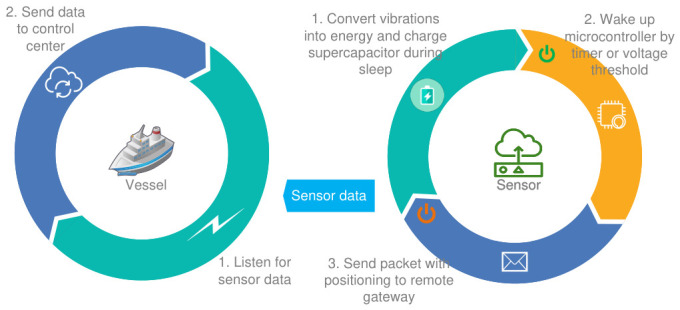
Detection process flowchart.

Within the waste detection stage, as illustrated in Figure 3, it is assumed that an energy harvester affixed to the bottom of a bottle transduces wave-induced vibrations into electrical energy. The resulting alternating current output is subsequently rectified and elevated in voltage using a DC-DC converter to charge a small supercapacitor. An energy management integrated circuit continuously monitors the stored voltage; upon reaching a predefined threshold (e.g., 3.6 V), the circuit activates an ultra-low-power microcontroller, which briefly energizes a positioning sensor. Following successful location identification, the microcontroller transmits a data message to a remote gateway before immediately reverting to the deep-sleep state. The supercapacitor is recharged by persistent wave fluctuations, and this operational cycle repeats indefinitely at intervals of a few minutes, thereby enabling maintenance-free, battery-less monitoring of waste fields.

In the proposed system we advocate to utilize the cap to embed a transmitting device. The rationale is that, as of July 2024, the EU mandates that all single-use plastic beverage containers up to 3 L must have caps that remain attached (tethered caps) during use, as mandated by Directive (EU) 2019/904 to reduce plastic waste. The initiative, focused on eco-design, aims to prevent plastic caps from being discarded separately, fostering a circular economy by increasing recycling rates of both the bottle and cap together.

**Figure 4:**
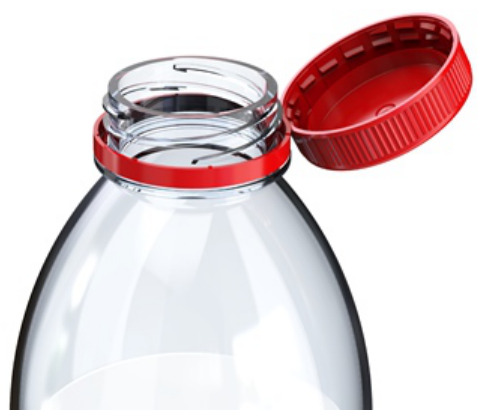
Illustration of the tethered cap which meets the EU’s Single-Use Plastics Directive [34].

An example of such a “tethered ” cap is shown in Figure 4. As of 2025, it is universally utilized across the EU. This directive allows a simple and straightforward application of the proposed approach, where electronic components are incorporated into the bottle caps, gathered together with the bottles, and not lost during recycling. The preliminary results reported in, e.g., [32,33], show that in spite of obstacles from private-owned companies, the initiative has been successful expecting the worldwide adoption in the coming decades.

Note that one may consider Radio Frequency Identification (RFID) as a potential technology for the considered purpose. However, RFID also includes medium-access protocols that allow the arbitration of connection requests from different end nodes. In our application, we do not need to do this, as simply hearing the emitted signal at the given frequency is sufficient. Thus, the end node should represent itself as an energy harvester and the transceiver part implements no logic at all.

**Figure 5:**
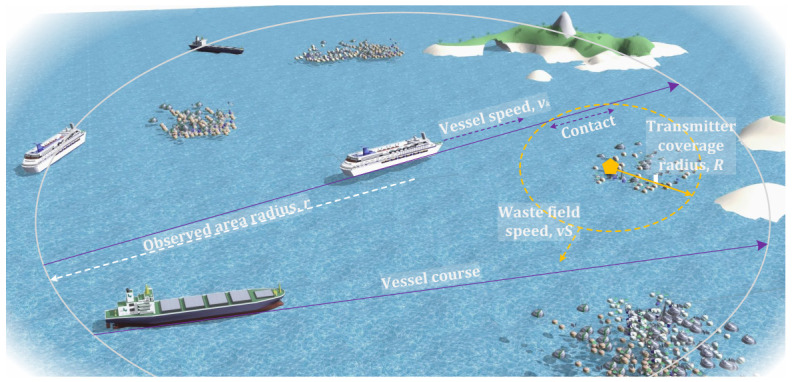
Illustration of the considered deployment.

The system is intended to operate as follows; see Figure 5. Note that this illustration provides interpretation of the considered system in terms of real entities such as ships, plastic fields, etc. Along their routes, vessels crossing a certain region of interest have active receivers. Once the carrier was detected, the coordinates were stored internally. The global system is presumed to be in operation, collecting and analyzing the stored coordinates. This system is expected to map the locations detected by various vessels and identify the locations of waste fields. Once completed, the waste removal techniques specified in Section 2.4 are utilized. The overall system is expected to operate continuously, with waste removal at regular intervals. We also note that the proposed system can coexist with other techniques for detecting waste fields in the ocean, as shown in Section 2.3.

Finally, we also stress that there has to be some incentives provided by the government to shipping companies for installing the detection system and passing the result to governmental agencies. There might be several ways to enforce this, ranging from licensing of operations to fees introduced. However the specific approach is of economic nature and thus out of the scope of our study.

### 3.2. The Modeled System

We consider a circularly shaped part of the ocean with radius *r*, as shown in Figure 5. We assume that there are *N* plastic waste fields, resulting in a density of $\lambda_{P}=N/\pi r^{2}$ fields/km^2^. The plastic waste fields are assumed to move according to the random direction model (RDM) [35]. According to this, a direction is first chosen randomly and uniformly in (0,2π). The field then moves in the chosen direction for an exponentially distributed time with parameter $1/E[\tau_{S}]$ at a constant speed $v_{S}$. This movement can be caused by water streams in the wind [36,37]. The waste fields are expected to be homogeneous; that is, the density $\lambda_{P}$ is constant. This implies that the flow of fields through the boundary of the zone is constant.

Each field is assumed to contain *M* plastic wastes equipped with the proposed notification system. The coverage radius of each radio is *R* km. Each radio is assumed to send a packet every Δt s. Ships/vessels are assumed to be equipped with receivers that constantly operate in the receive-ready mode. Vessels enter the considered zone with intensity $\lambda_{V}$ vess./h. The vessels are assumed to follow a random chord path through the considered zone. The speed is assumed to be a constant $v_{K}$.

We assume that if a vessel is currently in the coverage area of the transmitter and the transmitter generates a message, this message is successfully received and decoded by the receiver. In this section, we are interested in the probability that all plastic waste fields will be detected in time *T*, $p_{D,N}\left(T\right)$. Once a waste field is detected, any removal method can be used for cleanup, as described in Section 2.4.

### 3.3. Metrics of Interest

The ultimate metric of interest is the time that *i* waste fields are detected in a given time, with the special case that all the fields are detected in a given time. We derived this metric by first considering the contact probability between a single vessel crossing a region of interest and then extending it to the case in which a single field is detected. Finally, the sought probability is obtained by extending the derivations to the case of multiple waste fields.

## 4. Performance Analysis

**Figure 6:**
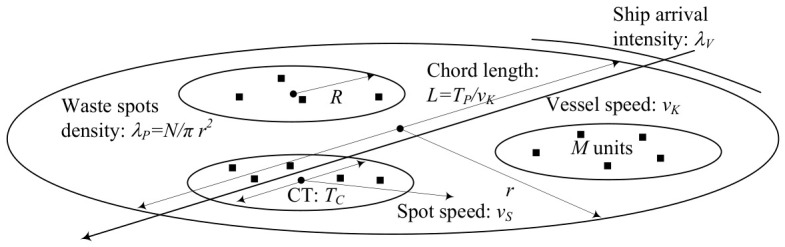
Abstracted view of the considered deployment for performance analysis.

In this section, we analyze the proposed pollution detection system by deriving the time-dependent metrics of interest identified in the previous section. Specifically, we will begin with the contact probability, then convert it to the detection probability, and finally derive the metrics specifying the probability of waste field detection in a given time. For further exposure in this section, we refer to the schematic diagram of the considered deployment, as shown in Figure 6. This illustration provides an abstracted view of the scenario illustrated in Figure 5 interpreting entities in terms of a technical system model.

We specifically note that, due to the absence of ready-to-use prototype, the model developed in this section is qualitative in nature. This allows us to evaluate the principal capabilities of the proposed system. However, it does not report on concrete quantitative values of performance measures that can be observed in realistic deployments.

### 4.1. Contact Probability

First, consider a single vessel crossing the considered zone. We are interested in the probability that this vessel, upon crossing the zone, will detect exactly *i* waste fields. To determine this, we need (i) the probability that a vessel crosses exactly *i* waste fields and (ii) while crossing each of those, it actually hears the transmitted message.

Let us first consider the former probability. Following [35,38] the point moving according to the RDM in a closed compartment is uniformly distributed in this compartment, that is, $f\left(x,y\right)=1/\pi r^{2}$. The probability density function (pdf) of the random chord length *L* specifying the vessel paths through the zone is given by [39]. Note that a random chord can be defined using multiple ways; see [39]. Here, we assume that the random chord is defined by two randomly distributed point at the circumference.

(1) $$\begin{array}{c} f_{L}\left(x\right)=\frac{2}{\pi \sqrt{4r^{2}-x^{2}}},\;0\leq x\leq 2r. \end{array}$$

(2) $$\begin{array}{c} \phi_{i}\left(T_{C}\right)=\left\{\begin{array}{cc} \frac{1}{\pi }\cos^{-1}\left(\frac{-2Rv_{K}\left(v_{S}^{2}+v_{K}^{2}\right)T_{C}+2v_{S}v_{K}T_{C}\sqrt{4R^{2}v_{K}^{2}-{\left(v_{S}^{2}-v_{K}^{2}\right)}^{2}T_{C}^{2}}}{4v_{K}^{2}\left(R^{2}+v_{S}^{2}T_{C}^{2}\right)}\right),\; & 0\leq T_{C}\leq \left|\frac{2Rv_{K}}{v_{K}^{2}-v_{S}^{2}}\right|\\ \frac{1}{\pi }\cos^{-1}\left(\frac{-2Rv_{K}\left(v_{S}^{2}+v_{K}^{2}\right)T_{C}-2v_{S}v_{K}T_{C}\sqrt{4R^{2}v_{K}^{2}-{\left(v_{S}^{2}-v_{K}^{2}\right)}^{2}T_{C}^{2}}}{4v_{K}^{2}\left(R^{2}+v_{S}^{2}T_{C}^{2}\right)}\right),\; & 0\leq T_{C}\leq \frac{2Rv_{K}}{v_{K}^{2}+v_{S}^{2}}\\ -\frac{1}{\pi }\cos^{-1}\left(\frac{-2Rv_{K}\left(v_{S}^{2}+v_{K}^{2}\right)T_{C}-2v_{S}v_{K}T_{C}\sqrt{4R^{2}v_{K}^{2}-{\left(v_{S}^{2}-v_{K}^{2}\right)}^{2}T_{C}^{2}}}{4v_{K}^{2}\left(r^{2}+v_{S}^{2}T_{C}^{2}\right)}\right),\; & \frac{2Rv_{K}}{v_{K}^{2}+v_{S}^{2}}\leq T_{C}\leq \left|\frac{2Rv_{K}}{v_{K}^{2}-v_{S}^{2}}\right| \end{array}\right.. \end{array}$$

To proceed further, we need the elements of the integral geometry utilized for similar problems; see, for example, refs. [40,41,42]. The following two definitions are provided.

**Kinematic density [43]**. Let *K* denote the group of motions of a set *A* in a plane. The kinematic density dA for the group of motions *K* in the plane for the set *A* is

(3) $$\begin{array}{c} dA=dx\wedge dy\wedge d\phi , \end{array}$$

where ∧ is the exterior product [44], *x* and *y* are Cartesian coordinates, ϕ is the rotation angle of *A* with respect to OX.

**Kinematic measure [43]**. The kinematic measure *m* of a set of group motions *K* on the plane is defined as the integral of the kinematic density dA over *K*, that is,

(4) $$\begin{array}{c} m_{A}=\int_{K}dA=\int_{K}dx\wedge dy\wedge d\phi . \end{array}$$

By further following [43] the probability that a finite segment *L* of length *l*, fully contained in the convex set $K_{0}$, crosses another convex set *K* also fully contained in $K_{0}$ is given by

(5) $$\begin{array}{c} \frac{m(L;L\cap K\neq 0)}{m(L\in K_{0})}=\frac{2\pi F+2lW}{m(L\in K_{0})}, \end{array}$$

where *F* and *W* are the area and perimeter of *K*.

Since in our case both $K_{0}$ and *K* are of circular shapes, $F=\pi R^{2}$, W=2πR, while the kinematic measure in the denominator are provided by

(6) $$\begin{array}{cc} m(L\in K_{0}) & =0.5\pi [2\pi r^{2}-8r^{2}\sin^{-1}\left(\frac{l}{2r}\right)-\\ \; & -2l\sqrt{4r^{2}-l^{2}}]. \end{array}$$

Substituting (6) into (5) and accounting for randomness of the vessel path through the considered zone in (1) we arrive at the following probability than a single vessel crosses a single randomly and uniformly distributed waste field

(7) $$\begin{array}{c} \;p_{C,1}=\int_{0}^{2r}\frac{f_{L}\left(x\right)\pi^{-1}\left(4\pi^{2}R^{2}+8x\pi R\right)}{2\pi r^{2}-8r^{2}\sin^{-1}\left(x/2r\right)-2x\sqrt{4r^{2}-x^{2}}}dx, \end{array}$$

which can be evaluated numerically for any *r* and *R*.

Now recalling that waste field are uniformly distributed in the considered zone, the probability that a single vessel will be in contact with *i* out of *N* fields is given by Binomial distribution with parameters $p_{C,1}$, i.e.,

(8) pC,i=NipC,1i(1−pC,1)N−i.

### 4.2. Detection Probability

Note that being in the coverage area of the radio of waste field *i* does not guarantee that this spot will be detected. We now proceed to evaluate the probability that a spot will be detected. To this aim, we first determined the contact time (CT) a vessel spends within the coverage of radios from a waste field. Waste fields are assumed to move according to the RDM, while vessels cross the area along straight lines. Assuming that the runtime of a waste field in a certain direction $v_{S}E\left[\tau_{s}\right]$ is much longer than the contact time, the sought metric can be obtained by considering both waste fields and vessels moving along straight trajectories.

Note that a vessel and waste field come in contact with each other only when the distance between them is less than *R*. Given that at the initial instant of time, the vessel is located on the circumference of the coverage area of a waste field is directed toward it, one needs to establish the CT distribution, $f_{T_{C}}\left(t\right)$, that is, the duration that the vessel spends before coming in contact with the circumference again.

Let the waste field at the beginning of the CT, that is, at $t_{0}$ be located at the origin, $x_{0}=0$ and $y_{0}=0$, and assume that the user is at a distance of *R* from it, at the coordinates $x_{U}=0$ and $y_{U}=l$. The waste field moves with the speed of $v_{S}$ along the horizontal axis, and the vessel moves at the speed of $v_{K}$ making an angle α with the *x*-axis. We are thus interested in the CT $T_{C}$, which satisfies the circle equation

(9) $$\begin{array}{c} {\left(x-x_{0}\right)}^{2}+{\left(y-y_{0}\right)}^{2}=R^{2}, \end{array}$$

where *x* and *y* are the coordinates of the vessel.

Further, the coordinates of vessel dynamics over time are

(10) $$\begin{array}{c} x=x_{V}+T_{C}v_{K}\cos\left(\alpha \right),\;y=y_{V}+T_{C}v_{K}\sin\left(\alpha \right). \end{array}$$

(11) $$\begin{array}{c} f_{T_{C}}\left(t\right)=\frac{1}{\pi }\frac{d\cos^{-1}\left[\frac{-2Rv_{K}\left(v_{S}^{2}+v_{K}^{2}\right)t+2v_{S}v_{K}t\sqrt{4R^{2}v_{K}^{2}-{\left(v_{S}^{2}-v_{K}^{2}\right)}^{2}t^{2}}}{4v_{K}^{2}\left(R^{2}+v_{S}^{2}t^{2}\right)}\right]}{dt}+\frac{1(t)}{\pi }\frac{d\cos^{-1}\left[\frac{-2Rv_{K}\left(v_{S}^{2}+v_{K}^{2}\right)t-2v_{S}v_{K}t\sqrt{4R^{2}v_{K}^{2}-{\left(v_{S}^{2}-v_{K}^{2}\right)}^{2}t^{2}}}{4v_{K}^{2}\left(R^{2}+v_{S}^{2}t^{2}\right)}\right]}{dt}. \end{array}$$

Substituting (10) into (9), we arrive at

(12) $$\begin{array}{c} {\left(T_{C}v_{K}\cos\left(\alpha \right)-T_{C}v_{S}\right)}^{2}+{\left(R+T_{C}v_{K}\sin\left(\alpha \right)\right)}^{2}=R^{2}. \end{array}$$

Solving (12) with respect to the CT gives us the following. Note that (13) is not defined for a very specific case of perfectly aligned speeds and directions, that is, $v_{S}=v_{K}$ and cos(alpha)=1. Specifically, the denominator $2v_{S}v_{K}\cos\left(\alpha \right)-v_{S}^{2}-v_{K}^{2}$ vanishes whenever $2v_{S}v_{K}\cos\left(\alpha \right)=v_{S}^{2}-v_{K}^{2}$, which reduces to the case $v_{S}=v_{K}$ with cos(α)=1 only because that is the unique real solution.

(13) $$\begin{array}{c} T_{C}=\frac{2Rv_{K}\sin\left(\alpha \right)}{2v_{S}v_{K}\cos\left(\alpha \right)-v_{S}^{2}-v_{K}^{2}}. \end{array}$$

As the vessel comes in contact with the waste field coverage at an arbitrary random angle α, the latter follows a uniform distribution in (0,π) with the associated density $f_{\alpha }\left(x\right)=1/\pi$. By employing random variable transformation techniques [45,46], the density of the CT can be expressed in an explicit form. Denote the direct transformation $T_{C}=f\left(\alpha \right)$, where f(α) is the right-hand side of (13). Further, we require the inverse transform $\alpha =\phi (T_{C})$, which is the solution to the following quadratic equation with respect to cos(α)

(14) $$\begin{array}{cc} \; & 4v_{K}^{2}\left(T_{C}^{2}v_{S}^{2}+R^{2}\right)\cos^{2}\left(\alpha \right)-4rv_{K}T_{C}\left(v_{K}^{2}+v_{S}^{2}\right)\cos\left(\alpha \right)-\\ & \;-4v_{K}^{2}v_{S}^{2}T_{C}^{2}-T_{C}\left(v_{K}^{2}+v_{S}^{2}\right)=0. \end{array}$$

Observing that 0≤α≤π, the inverse transform in question has three branches, which leads to the following pdf of the transform $T_{C}=f\left(\alpha \right)$

(15) $$\begin{array}{c} f_{T_{C}}\left(y\right)=\sum_{1\leq i\leq 3}f_{\alpha }\left(\phi_{i}\left(y\right)\right)\left|\frac{d\phi_{i}\left(y\right)}{dy}\right|. \end{array}$$

The resulting pdf of $T_{C}$ is presented in (11), where $0\leq t\leq |2Rv_{K}/\left(v_{K}^{2}-v_{S}^{2}\right)|$. Note that in the special case of $v_{S}=0$, that is, when the speed of waste fields is negligible compared to the speed of vessels, the CT reduces to

(16) $$\begin{array}{c} f_{T_{C}}\left(x\right)=\frac{2v_{K}}{\pi \sqrt{4R^{2}-{\left(xv_{K}\right)}^{2}}},\;0\leq x\leq 2Rv_{K}. \end{array}$$

**Figure 7:**
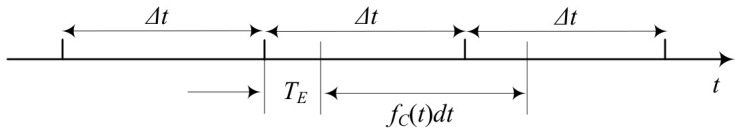
Time diagram of detection probability.

Once $f_{T_{C}}\left(t\right)$, t>0, is found, we are in a position to determine whether the transmission will be detected given that. Recall that as the notification systems are assumed to be non-synchronized, it is sufficient to determine the probability that we receive a transmission from a single transmitter, $q_{D}$. To this end, consider Figure 7, where Δt is the inter-message time interval, $T_{E}$ is the time between previous message and the beginning of the contact time. In order for a vessel to detect the waste field, the following has to be satisfied

(17) $$\begin{array}{c} \;q_{D}=Pr\left\{T_{E}+T_{C}\geq \Delta t\right\}=Pr\left\{T_{E}+T_{C}-\Delta t\geq 0\right\}. \end{array}$$

Observe that $T_{E}$ has a uniform distribution over (0,Δt) with pdf $f_{T_{E}}\left(t\right)=1/\Delta t$. Furthermore, random variables $T_{E}$ and $T_{C}$ are independent from each other implying that the pdf of their sum can be obtained by the convolution technique, i.e.,

(18) $$\begin{array}{c} f_{T_{E}+T_{C}}\left(t\right)=\int_{0}^{\infty }f_{T_{E}}\left(\tau \right)f_{T_{C}}\left(t-\tau \right)d\tau . \end{array}$$

Finally, the component −Δt causes the displacement of the pdf over *x*-axis, that is, $f_{T_{E}+T_{C}-\Delta t}\left(t\right)=f_{T_{E}+T_{C}}\left(t+\Delta t\right)$.

Now the probability that the waste field will be detected during the CT between vessel and a waste field is given by

(19) $$\begin{array}{c} q_{D}=\int_{0}^{\infty }f_{T_{E}+T_{C}-\Delta t}\left(t\right)dt. \end{array}$$

Once the conditional detection probability $q_{D}$ is obtained, the probability that a single vessel detects *i* out of *N* waste fields in the considered area is given by Binomial distribution with parameter $q_{D}p_{C,1}$

(20) pD,i=Ni(pC,1qD)i(1−pC,1qD)N−i.

### 4.3. Detection in a Given Time

We now proceed to provide the probability that *i* out of *N* waste fields are detected in time *T*. Let $T_{P}$ be the passage time of a single vessel through the considered area. $T_{P}$ is a random variable whose distribution is a scaled version of $f_{L}\left(x\right)$ obtained in (1), that is, $f_{T_{P}}\left(t\right)=f_{L}\left(xv\right)$, 0≤t≤2r/v. Having this distribution at our disposal, we determine the number of vessels crossing the considered area in time *T* as $H=\lfloor T/T_{P}\rfloor$. Note that the pdf of $T/T_{P}$ can be obtained by applying random variable discretization technology as shown below

(21) $$\begin{array}{c} f_{T/T_{P}}\left(i\right)=\frac{\int_{iT}^{(i+1)T}f_{L}\left(xv\right)dx}{T},\;i=0,1,2,\ldots . \end{array}$$

Once $f_{T/T_{P}}\left(x\right)$ is obtained, we utilize a discretization operation to produce the probability mass function (pmf), $g_{i}$, i=0,1,…, where $g_{i}$ is the probability that exactly *i* vessels pass through the considered area in time *T*. Now, the probability that *i*-th waste field will be detected by at least one vessel is

(22) $$\begin{array}{c} p_{D,1}\left(T\right)=\sum_{i=1}^{\infty }g_{i}\left[1-{\left(1-p_{C,1}q_{D}\right)}^{i}\right]. \end{array}$$

Now, the probability that exactly *i* waste fields will be detected is

(23) pD,i(T)=NipD,1(T)i[1−pD,1(T)]N−i,

that produces the probability that at least *j* waste fields will be detected in the following form

(24) $$\begin{array}{c} p_{D,\leq j}\left(T\right)=1-\sum_{i=0}^{j}p_{D,i}\left(T\right), \end{array}$$

leading to $p_{D,N}\left(T\right)$ when j=N.

## 5. Energy-Harvesting System

In this section, we first outline the principles of energy harvesting (EH). Then, we provide an overview of techniques that can be used to enable EH at extremely low vibration frequencies, which are typical of oceans. Finally, we briefly describe a prototype that can be used for the system considered in this study.

### 5.1. Energy-Harvesting Principles

**Figure 8:**
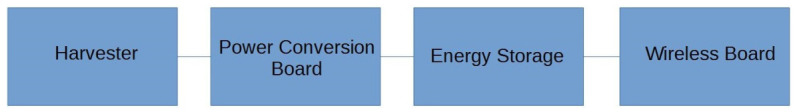
Structural diagram of the wireless node powered by energy harvester.

Energy-harvesting processes usually consist of three main steps: (i) accumulating energy–process to be done with energy harvester, (ii) storage of power–in case it is required by application, (iii) use of power–main goal of the process and the determinant of the efficiency. The structural diagram of a wireless node powered by EH is shown in Figure 8 [47,48].

EH leverages diverse natural and ambient sources through distinct transduction mechanisms, each with specific operational requirements. Magnetostrictive harvesting is particularly suitable for structural monitoring of massive metal installations, such as bridges and communication towers, although it necessitates high precision in material selection and structural design [49]. In contrast, inductive harvesters utilize mechanical kinetic energy from sources such as wind or vibration to provide effective, autonomous power, whereas piezoelectric systems offer high efficiency but are constrained by the specific amplitude and directional alignment of the mechanical excitation [50]. Solar harvesting remains a highly effective method for achieving high-power yields; however, its utility is strictly dependent on consistent light exposure and periodic maintenance of photovoltaic surfaces. In addition to these primary methods, several alternative transduction techniques have been extensively documented in the literature, expanding the versatility of self-powered systems across various industrial applications [51].

In our study, we chose two main methods to be tested: magnetostrictive and inductive. These methods do not require special conditions for the harvester to work and are almost completely independent of the environment. In addition, with these methods, a power level of up to 50 mW is achievable, as required for the considered application.

### 5.2. Ultra-Low-Power Energy Harvesting

To keep up with the ever-growing volume of collected data and meet industrial needs for reliable and maintenance-free sensors, energy harvesting may become an indispensable solution. Currently, various studies have considered different ways of implementing EH and studied the possibilities and effectiveness of these processes [47,48]. Wireless industrial sensors may be an important, yet costly, data collection infrastructure. Most of these sensors require periodic maintenance owing to manually rechargeable battery power supplies. This means that with EH, it may be possible to abandon batteries if sufficient energy is available in the environment. The ocean provides a theoretically unlimited amount of energy, subject to the use of wind and waves.

The main research-intensive part of an EH system is the harvester and power converter board. In addition, there is no special product, such as a DC/DC boost converter, for low-power EH, and one of the goals of this study is to develop a working prototype of such a device. This will increase the number of use cases and accelerate the development of new solutions.

**Figure 9:**
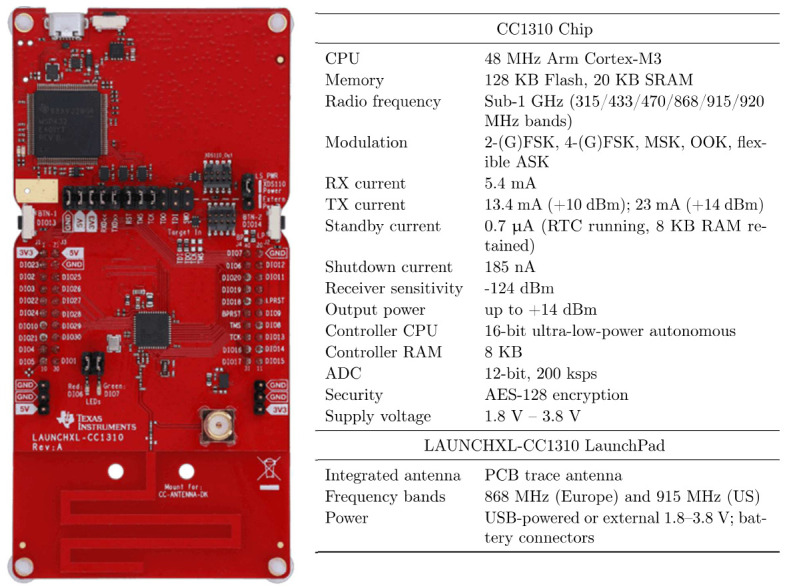
CC1310 LaunchPad LAUNCHXL-CC1310 chip.

As sensor networks and wireless communications are good scenarios for EH [52], it is important to consider a wireless node for its operation. This node usage provides empirical results and the necessary power levels of the energy harvested. Sub-GHz band wireless nodes provide energy-efficient wireless communication, which can be used with EH. As a perspective wireless board, a wireless microcontroller development kit for rapid prototyping based on a CC1310 chip produced by Texas Instruments Inc. (Dallas, TX, USA) was chosen (CC1310 LaunchPad LAUNCHXL-CC1310; Figure 9). This platform allows for low-frequency high-range wireless data exchange with low energy consumption (up to 50 mW with 3.3 V input voltage).

The absence of batteries allows work in low-temperature conditions and ensures a long lifetime of the system. Owing to the size of mechanical harvesters, it may be necessary to separate them from electronic devices. This will not present a major change in the overall robustness of the product (as there are no electronics in mechanical harvesters) but will provide flexibility in the implementation stage. The desired outcome of this study is to demonstrate the possibility of developing a low-cost and effective energy harvester.

### 5.3. System Prototyping

For the proposed system, one may be utilized. The main effect used in this process is electromagnetic induction, which generates current flow within the coil when there is a changing electromagnetic field nearby. Because the coil is the power source of our device, it is necessary to conduct several experiments to determine its energy characteristics.

Initially, widely available coils and neodymium magnets were used, but they did not provide high energy results (less than 10 mW per second was generated using vibrational EH). In addition, several types of cores were tested, but they did not provide sufficient results at low frequencies (below 3 Hz). The materials tested included paramagnetic, ferromagnetic, and diamagnetic metals, iron, and steel. The most predictable results and highest power levels were demonstrated when a high-force neodymium magnet moved inside a coreless coil.

Most existing materials for coil cores operate within a specific frequency range (see Table 3), indicating that for the selected application, the optimal solution is to use coreless coils for this type of EH. This is because of the challenge of using very low frequencies, which occur when mechanical EH from natural forces is generated. Natural forces such as waves or wind generate oscillations with low frequencies (approximately several hertz) but may produce a large amount of power (theoretically unlimited), and our goal is to harvest as much energy as possible from them.

There are multiple types of coils available in the market. However, for specific EH tasks, custom coils are required, especially in the research stage, as they can be adapted to create magnetic assemblies. The coil experiments showed that the minimum number of turns for a coil without a core was 500. One thousand turns on the coil showed applicable energy levels (hundreds of micro watts) with the load. More than 1000 turns coils are more difficult to make, so this amount of turns was taken as a baseline for all the final experiments. Most coil analyses have been performed empirically. Various types of magnets and their assemblies were tested to create a sufficiently powerful and at the same time compact mechanical harvesting system.

According to the tests conducted, it can be concluded that the energy characteristics of the coils are relatively small. The voltage amplitude of 2 V was maximum for a load of 2 kΩ. The 5 mA current in the low frequency mode was a compromise level for the harvester to work for the low-power applications. The chip used for the wireless node was a Texas Instruments CC1310 Ultra-Low-Power Sub-1 GHz Wireless microcontroller. The chip requires 11.2 mA with 3.6 V direct digital synthesis voltage for a standard transmission mode according to the official datasheet. This means that with a low-power voltage up-converter and energy storage, it will be possible to harvest enough energy with this setup for periodic wireless transmissions.

After conducting several experiments with cored coils, a neodymium magnet fluctuating within a coreless coil was chosen as the primary direction of work. It adopts the well-known effect of magnetic induction and allows the production of a relevant amount of current through the coil. The experiments demonstrated that the voltage peaks could not exceed 0.5 V. According to Ohm’s Law, power of the harvester is 125 μW, that is,

(25) P[2W]=((U[V])2/R[Ω])U[V]=125[μW],

where *P* is the power, *U* is the voltage, and *R* is the resistance.

Note that 125 μW with 0.5 V is not enough for any existing DC/DC converter to initialize and is also insufficient to power a wireless node that requires at least 50 mW. Therefore, we considered increasing the induction of the magnetic field and the number of coil turns. To increase the magnetic field force, a magnetic assembly of two magnets with an adhesive force of 200 kg each was used. When two magnets are located close to each other they are able to create very high gradient of magnetic field which in its turn allows the creation of higher currents in the coil with 1–2 mm movement. The main goal of this system is to generate the highest possible current with the smallest possible movement of the coil. Consequently, a peak voltage of was observed with a load of 2 kΩ. It provides around 4.5 W per oscillation, which is sufficient to power a wireless node.

Several types of mechanics have been used to achieve the required energy generation level. Several compliant mechanisms were created to test various vibration harvesters. Compliant frequency up-converters were developed to increase the oscillation frequency and produce the necessary flux within the coil. The first attempt was to create a highly vibrating spring to obtain high-frequency oscillations from very low-frequency vibrations. This system allows obtaining up to 10 Hz fluctuations from less than 1 Hz waves inside large metal structures. After several tests, it became evident that the current design of the compliant oscillator requires improvement. The main reason for using compliant mechanics is that they provide the possibility of creating a frequency up-converter. It is clear that a higher frequency than the commonly available up to several hertz is required for a higher efficiency of a mechanical energy harvester. In addition, it does not demonstrate a high conversion rate (up to ×3 frequency conversion, but with a high fading effect) and requires operation under special conditions (the oscillation working plane is fixed). The main idea for energy generation in a mechanical energy harvester (based on the inductive principle) is to have a relatively heavy pendulum that moves a small coil in a strong magnetic field to generate energy. In practical system, this is supposed to emulate the ocean waves vibrations. The coil oscillation frequency within a typical application (for example, a tree swaying in the wind) cannot exceed 5 Hz. Such a low operational frequency may provide sufficient power only if each oscillation provides sufficient energy to start a DC/DC converter. The final weight of this construction was approximately 6 kg. The final step in the mechanical energy generator was the compliant mechanics effect using two hacksaw blades. The blades were used because they were springy and strong at the same moment. In addition, the blades allow compliant oscillations in the horizontal direction and do not move in the vertical direction. This system allowed us to have a mechanism similar to that of a lever arm, with a mechanical advantage of ten. Therefore, when the pendulum was moved 1 mm, the coil moved approximately 1 cm. Because the pendulum was very heavy compared with the coil, the losses were insignificant. Nevertheless, this energy is insufficient to perform a wireless transmission with only one oscillation. Thus, several high-capacity capacitors were adopted for the temporary storage of energy, with a total of 40 mF.

## 6. Numerical Results

In this section, we assess the performance of the proposed system. We specifically assess the impact of the considered area, number of waste fields in this area, radio coverage, and vessel intensity on the probability that *i* out of the available *N* waste fields are detected in a given time *T*, $p_{D,i}\left(T\right)$. The default parameters for the system evaluation are listed in Table 4. Note that throughout this section, the inter-message interval generated by the harvesting-based radios is set to 600 s.

Note that in this section we intentionally did not utilize a detailed channel model in our study and also tried to abstract the rest of the parameters. The rationale for this is that the developed model includes several critical assumptions that may not be verified precisely. This includes the propagation model that heavily depends on the state of the sea, the emitted power that is still unknown at this stage, and the mobility of waste fields. The analysis performed should not be considered as quantitative rather than a qualitative one. To this end, in Section 6, we cover a large range of various values of interest (excluding those that may duplicate each other’s impact, e.g., path loss and emitted power). Whenever possible we also abstracted these internal values with easily interpretable quantities.

**Figure 10:**
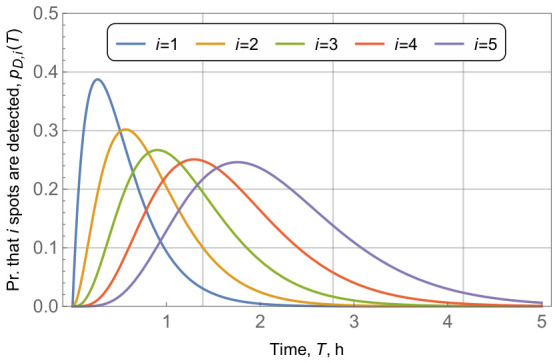
Pmfs of detecting *i* our of *N* waste fields in time, *T*, $p_{D,i}\left(T\right)=f\left(T\right)$, for multiple time intervals *T*.

We begin with the assessment of the pmfs of detecting *i* out of *N* waste fields in a certain time, *T*, for i=1,2,…,5, where the overall number of waste fields is N=10, area radius is r=10 km, radio coverage is R=r/5=2 km, and vessel intensity is $\lambda_{V}=3.6$ vess./h. By analyzing the data presented in Figure 10, one may observe that when the number of waste fields to detect *i* increases, the pmfs shift to the right, implying that the probability of detection decreases. However, the case of i=1 is of special interest, as detecting even a single waste field in the area is crucial. The rationale is that during the waste removal phase, the rest of the waste fields can be detected visually. For the selected parameters, the majority pmf mass for i=1 was concentrated within a single hour. The detection of more than a single waste field might be crucial for the overall assessment of the density of waste fields in the ocean and for prioritizing regions for waste removal. To increase this probability one may increase the radio coverage *R* of a single transmitter. However, an inherent trade-off exists between the EH, radio coverage, and board limitations.

**Figure 11:**
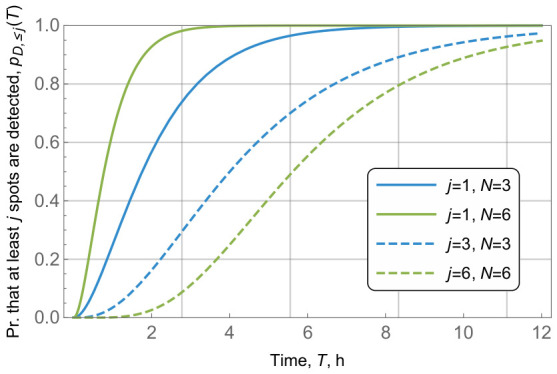
Probabilities that all and at least one waste spot is detected, $p_{D,N}\left(T\right)$, $p_{D,>1}\left(T\right)=f\left(T\right)$.

Having understood the importance of detecting at least one and all waste fields in the area, we now proceed to address these characteristics in detail. To this aim, Figure 11 illustrates two probabilities, $p_{D,N}\left(T\right)$ and $p_{D,\geq 1}\left(T\right)$ for the overall number of waste fields in the area of N=3,6, area radius of r=10 km and radio coverage of R=r/5=2 km. By analyzing the results, we first observe that under given parameters, the probability that at least one waste field is detected approaches 1 in just 4 h. For the area with N=3 the detection of all waste spots also takes slightly more than 4 h. However, when *N* increases, the time for the detection of all waste fields increases.

**Figure 12:**
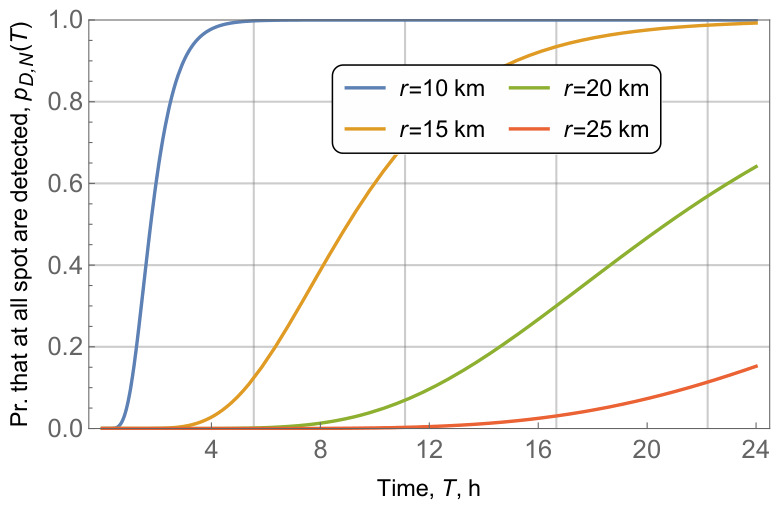
Probability that all waste fields are detected, $p_{D,N}\left(T\right)=f\left(T\right)$ for different values of area radius *r*.

We now proceed to explore the impact of additional system parameters, including the area radius, *r*, radio coverage, *R*, and vehicle intensity in the area, $\lambda_{V}$. We begin with the former parameter, whose impact on the probability that all waste fields are detected in the area is shown in Figure 12 for radio coverage R=2 km, number of waste fields in the area, N=10, intensity of vessel arrivals of $\lambda_{V}=10^{-3}$ vess./h. By analyzing the results shown in Figure 12, one may observe that area radius greatly impacts the the considered detection probability. Specifically, for r=10 km, all 10 waste fields are detected in approximately 6 h. However, when it increased to 25 km, the probability barely reached 0.2 in 24 h.

**Figure 13:**
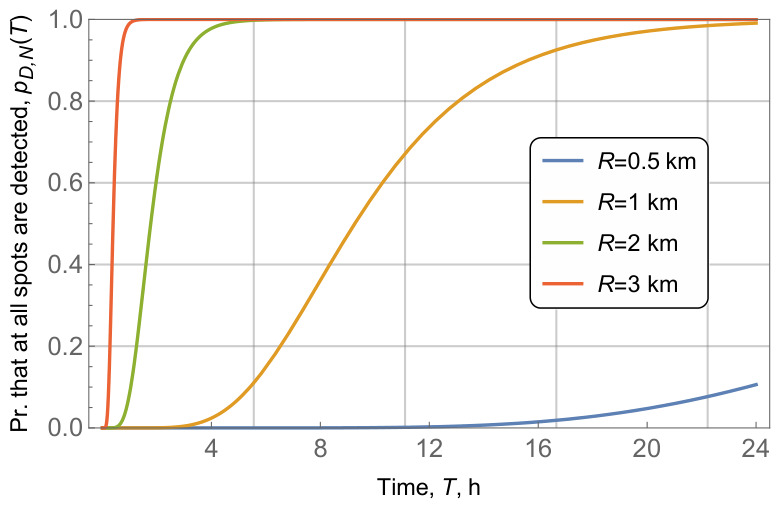
Probability that all waste fields are detected, $p_{D,N}\left(T\right)=f\left(T\right)$, for different values of radio coverage *R*.

We now turn our attention to the impact of radio coverage, *R*. To this end, Figure 13 demonstrated the probability that all waste fields are detected in a given time interval as a function of radio coverage *R*, for fixed value of area radius r=10 km, N=10 waste fields in the area, and vessel intensity of $\lambda_{V}=3.6$ vess./h. The results show that the impact of radio coverage *R* is similar to the area radius *r*. That is, relatively small values of *R* (R=0.5,1 km) lead to extremely small values of time to detect all the waste fields in the area, while increasing *R* to 2 and 3 km, increases the time to 24 h and more. That is, in the case of R=3 km, the probability of detecting all the waste fields in 24 h barely reaches 0.2.

**Figure 14:**
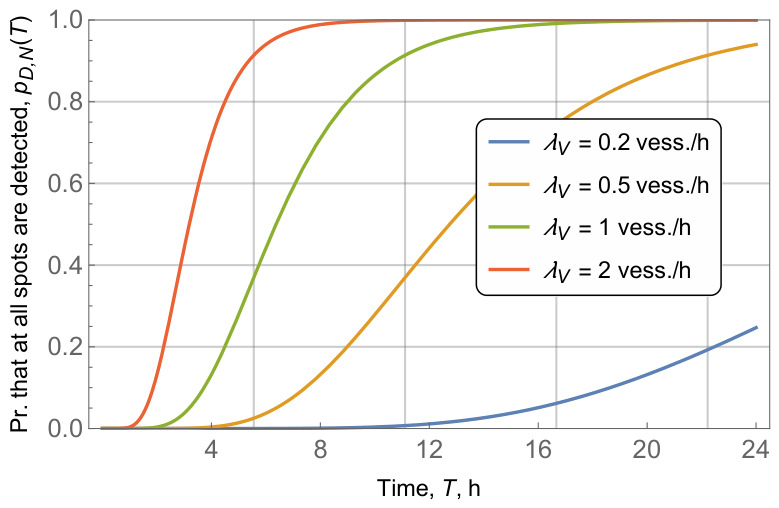
Probability that all the waste fields are detected, $p_{D,N}\left(T\right)=f\left(T\right)$, for different vehicle intensities $\lambda_{V}$.

The final parameter of interest is the vessel intensity, $\lambda_{V}$ whose impact on the probability that all waste fields in the area are detected is shown in Figure 14. The remaining parameters are fixed, that is, N=10, r=10 km, r=R/2=2 km. It is important to note that, unlike the area radius, *R*, and radio coverage, *r*, the vessel intensity is a system parameter that can be controlled by equipping more vessels with detection radios. As shown in Figure 14, the impact of $\lambda_{V}$ is quite drastic, that is, decreasing the vessels’ intensity by 10 times, from 2 vess./h to 0.2 vess./h leads from the probability of detecting all the waste fields in just 6 h to having a probability of detection of around 0.2 in 24 h. Even for rather small values of $\lambda_{V}$ the time for the detection of all waste fields is on the order of days.

## 7. Conclusions

Motivated by the increasing plastic pollution in oceans, we proposed, analyzed, and prototyped a plastic waste-field detection system. We employed low-frequency wave-powered radio transmitters in a subset of the plastic packages. The results show that in typical maritime traffic areas of 10–25 km^2^, at least one waste field can be detected within 24 h, and all fields within a few days. Detection probability depends largely on uncontrollable environmental factors but can be improved by increasing the share of receiver-equipped vessels. The proposed system can be used in conjunction with state-of-the-art waste field detection systems, such as satellite-based systems, to complement them. Additionally, it does not require novel waste removal systems and may rely on traditional ones.

We specifically note that in this paper we describe the principles of the energy-harvesting prototype we are currently working on. Although the prototype described in Section 5 is operational, its overall construction is still multiple kilos and is not appropriate for miniaturized devices that we target, where both the transceiver and harvester need to be of approximately bottle cap size to be included in tethered caps. Thus, one of the goals of this paper is to stimulate a new wave of research in building miniaturized prototypes for energy harvesting at low frequencies.

## Figures and Tables

**Table 1:** Sea-based marine plastic litter removal methods.

Method	Target Env.	Technology Readiness	Efficiency	Limitations
Manual beach cleanup (macropl.)	Shoreline, intertidal zone	Mature, widely deployed	Highly selective; labour-intensive	Ineffective for buried or microplastic debris; weather-dependent
Floating barrier/net system (macropl.)	Sea surface, coastal waters & oceanic gyres	Demo/early deployment (TRL 6–7)	Hundreds of tons removed from GPGP; periodic extraction required	Only floating debris; cannot collect sunken litter (94% of total); bycatch risk; high maintenance
Underwater robotic systems (macropl.)	Seabed, shallow coastal waters (<100 m)	Prototype/demo (TRL 5–6)	Positioning error <1 m; detection ≈80%; successful autonomous retrieval	High cost; limited depth; slow operation; not scalable to large areas; requires calm conditions
Manta trawl/sediment sieving (micropl. monitoring)	Sea surface or coastal seabed (sieving)	Research/monitoring only (TRL 4–5)	Not designed for remediation; low volume recovery	Not scalable for cleanup; high energy per particle; primarily for sampling
Bio-inspired/enzyme systems (micropl.)	Experimental (lab/controlled)	Early research (TRL 3–4)	High efficiency in lab (up to 95% under ideal conditions)	Not yet deployed in marine environment; stability and cost issues

**Table 2:** Notation utilized for performance modeling.

Notation	Description
*r*	Radius of the considered zone (km)
*N*	Number of plastic waste fields in the zone
$\lambda_{P}=N/\pi r^{2}$	Density of waste fields (fields/km^2^)
$E[\tau_{S}]$	Mean run length of a waste field in RDM (s)
$v_{S}$	Speed of the waste field (km/h)
*M*	Number of plastic waste with the proposed system
$\lambda_{V}$	Intensity of vessel arrivals (vess./h)
$v_{K}$	Speed of ships/vessels (km/h)
*L*	Random chord length (km)
$T_{P}$	Passage time of a vessel in the considered zone (km)
$T_{C}$	Contact time between the vessel and field (s)
*T*	Detection period (s)
Δt	Inter-message time interval (s)
d(·)	Kinematic density
$m_{\left(\;\cdot \;\right)}$	Kinematic measure
$f_{\left(\;\cdot \;\right)}\left(x\right)$	Probability density function
$F_{\left(\;\cdot \;\right)}\left(x\right)$	Cumulative distribution function
$p_{C,i}$	Probability that *i* vessels contact coverage of *j* fields
$p_{D,i}$	Probability that *i* vessels detect *j* waste fields
$p_{D,1}\left(T\right)$	Probability of waste field detection in time *T*
$p_{D,i}\left(T\right)$	Probability of *i* waste fields detection in time *T*
$p_{D,\leq j}\left(T\right)$	Probability of at least *i* waste fields detection
$q_{D}$	Conditional detection probability from
$p_{D}\left(T\right)$	Probability that all the fields are detected in time *T*

**Table 3:** Operating frequency ranges and key properties of common core materials

Core Material	Frequency Range	Key Properties
Laminated Silicon Steel	50–400 Hz	High saturation (1.5–2.1 T); low cost; high core losses above 400 Hz
Amorphous Steel	50 Hz–1 kHz	Lower core loss than Si steel; up to 1.56 T saturation; good efficiency
Iron Powder	10–200 kHz	Distributed air gap; moderate losses; good DC bias; permeability 10–100
Sendust (Fe-Si-Al)	20 kHz–2 MHz	Low loss (20% of iron powder); near-zero magnetostriction; up to 140k permeability
Ferrite (MnZn)	1 kHz–1 MHz	High permeability (750–15,000); low cost; moderate saturation (0.3–0.5 T)
Molypermalloy (MPP)	Up to 1 MHz	Low core loss; high permeability (14–550); excellent DC bias; expensive
Nanocrystalline	10 kHz–5 MHz (ext. to 30 MHz)	Very low loss; high permeability (up to 150k); compact size; up to 1.25 T saturation
Carbonyl Iron	1–100 MHz	High stability; excellent Q (50 kHz–200 MHz); high resistivity; low permeability (1–35)
Ferrite (NiZn)	1–300 MHz	High resistivity; low permeability (10–1000); good for high frequencies
Air Core	1 GHz	No saturation; lowest losses; zero permeability; low inductance per turn

**Table 4:** Default system parameters.

Notation	Description	Value
*r*	Radius of the considered zone (km)	10
*R*	Vessel coverage radius (km)	2
*N*	Number of plastic waste fields in the zone	10
Δt	Inter-message time interval (s)	600
$\lambda_{V}$	Intensity of vessel arrivals (vess./h)	3.6
$v_{K}$	Speed of ships/vessels (km/h)	10

## Data Availability

Dataset available on request from the authors.

## Abbreviations

The following abbreviations are used in this manuscript:

AI	Artificial Intelligence
CT	Contact Time
DC/DC	Direct Current to Direct Current
EH	Energy Harvesting
FDI	Floating Debris Index
HDPE	High-Density Polyethylene
HSI	Hyperspectral Imaging
IoT	Internet of Things
LCA	Life Cycle Assessment
MSI	Multispectral Imaging
PET	Polyethylene Terephthalate
POPs	Persistent Organic Pollutants
RDM	Random Direction Model
RGB	Red-Green-Blue
RS	Remote Sensing
SAR	Synthetic Aperture Radar
TIR	Thermal Infrared
TRL	Technology Readiness Level
UAS	Unmanned Aerial Systems
UNEP	United Nations Environment Programme
pdf	probability density function
pmf	probability mass function
